# Radiomics Texture Analysis of Bone Marrow Alterations in MRI Knee Examinations

**DOI:** 10.3390/jimaging9110252

**Published:** 2023-11-20

**Authors:** Spiros Kostopoulos, Nada Boci, Dionisis Cavouras, Antonios Tsagkalis, Maria Papaioannou, Alexandra Tsikrika, Dimitris Glotsos, Pantelis Asvestas, Eleftherios Lavdas

**Affiliations:** 1 Medical Image and Signal Processing Laboratory, Department of Biomedical Engineering, University of West Attica, 12241 Athens, Greece; cavouras@uniwa.gr (D.C.); pasv@uniwa.gr (P.A.); 2 Department of Biomedical Sciences, University of West Attica, 12241 Athens, Greece; nadia.botsi@gmail.com (N.B.); llavdas@uniwa.gr (E.L.); 3 Department of Radiology, Animus Kyanous Stavros, 57014 Larissa, Greece; 4 Department of Orthopedic, Animus Kyanous Stavros, 57014 Larissa, Greece; 5 Department of Radiology, General University Hospital of Larissa, 41334 Larissa, Greece; altsikrika@hotmail.com

**Keywords:** bone marrow edema, knee injury, osteoarthritis, radiomics, texture analysis

## Abstract

Accurate diagnosis and timely intervention are key to addressing common knee conditions effectively. In this work, we aim to identify textural changes in knee lesions based on bone marrow edema (BME), injury (INJ), and osteoarthritis (OST). One hundred and twenty-one MRI knee examinations were selected. Cases were divided into three groups based on radiological findings: forty-one in the BME, thirty-seven in the INJ, and forty-three in the OST groups. From each ROI, eighty-one radiomic descriptors were calculated, encoding texture information. The results suggested differences in the texture characteristics of regions of interest (ROIs) extracted from PD-FSE and STIR sequences. We observed that the ROIs associated with BME exhibited greater local contrast and a wider range of structural diversity compared to the ROIs corresponding to OST. When it comes to STIR sequences, the ROIs related to BME showed higher uniformity in terms of both signal intensity and the variability of local structures compared to the INJ ROIs. A combined radiomic descriptor managed to achieve a high separation ability, with AUC of 0.93 ± 0.02 in the test set. Radiomics analysis may provide a non-invasive and quantitative means to assess the spatial distribution and heterogeneity of bone marrow edema, aiding in its early detection and characterization.

## 1. Introduction

It is thought to be possible to predict pain, disability, and the structural progression of knee osteoarthritis using bone marrow lesions (BMLs). However, the relationship between knee loading and BMLs is not yet completely understood [1]. Moreover, the pathophysiology of osteoarthritis (OA) is also incompletely understood [2]. For example, the pathologic processes, primarily located at the level of the articular cartilage, do not completely characterize numerous OA findings. This includes bone marrow edema (BME) in the subchondral bone, detected via magnetic resonance imaging (MRI) in the affected joints [3]. Felson et al. defined a relationship between knee pain and bone marrow lesions using MRI [4], where bone marrow lesions on MRI were present in 78% of patients with painful OA knee compared to only 30% of patients with painless OA knee. Specifically, the degree of bone edema appeared to be associated with the severity of angular deformity at the knee [5,6].

There is evidence that subchondral BMLs imaged using MRI are involved in the pathogenesis of OA [4,5,7,8]. BME pattern is defined as an area of high signal intensity in T2- weighted, fat-saturated MRI or in short-inversion-time inversion recovery (STIR) images [9,10,11]. This increase in signal has been attributed to several factors, including abnormal trabeculae, bone marrow necrosis, swelling of fat cells, and marrow bleeding. It indicates a so-called bone bruise or impression of fracture due to translational injury, where the anterolateral femur impacts the posterolateral tibia (kissing contusions) when the ACL is ruptured [10]. Regarding healthy subjects with no OA, traumatic injuries of the knee can result in subchondral bone edema lesions [12,13]. However, the causal relationship between these lesions and the disease’s progression is unclear [8]. A better understanding of the interrelation of cartilage and the bone immediately under is necessary [14].

MRI-based texture analysis has been previously proposed for quantification of bone related abnormalities. Fritz et al. [15] and Cilengir et al. [16] have highlighted the diagnostic performance of texture analysis for cartilaginous bone tumors. Fritz et al. found 92.9% accuracy in differentiating benign from malignant cartilaginous lesions when employing both MRI and textural predictors. Cilengir et al. found an AUC of 0.84 in differentiating chondrosarcomas from encondromas employing the logistic regression classifier. Chuah et al. [17] performed texture analysis in the femur for the detection of cartilage-related bone marrow edema. They reported an AUC of 0.91 in differentiating normal from affected bone marrows lesions based on co-occurrence matrix features. Mackay et al. [18] used texture analysis for tibial subchondral bone assessment in OA. They reported 53% accuracy in differentiating between normal and OA cases based on statistical texture analysis. Recently, Li et al. used texture analysis of the infrapatellar fat pad for OA prediction [19]. They reported an AUC ≥0.75 in predicting OA progression employing textural features.

To the best of our knowledge, the utilization of texture analysis to differentiate between bone marrow edema, injury, and osteoarthritis lesions has not yet been reported. We chose to study these three clinical conditions, because they are very common in the knee joint and their radiological findings are not easy to distinguish. Sometimes, even when attempted in combination with clinical findings in patients with OA and injury, or in young patients with injury, it is difficult to make a specific diagnosis. However, such clinical cases require different clinical management, so it is important to differentiate, i.e., while bone marrow edema cases require no treatment, the injured and osteoarthritis cases require different clinical management. Hence, the present study is focused on studying the variations in those descriptors in the different knee abnormalities caused by bone marrow edema, injury, and osteoarthritis. For this, we considered a large pool of radiomics descriptors encoding texture information. These descriptors can express significant properties in the bone marrow edema, like lesions that might be useful in diagnosis, prognosis, or the treatment of individual patients [20].

## 2. Materials and Methods

### 2.1. Clinical Material

In the present study, one hundred and twenty-one individuals (121) were routinely scanned for knee examination from 2015 to 2017. This amounted to seventy females and fifty-one males with average age of 41 years. Non-selection criteria were subjects likely to have bone edema (multiple myeloma, Pazet disease, history of sarcoma, or known cancer showing secondary bone metastases).

The subjects were divided into three groups according to radiological findings [21]. The most dominant radiological findings are those which are related with the placement of the edema and its presentation (single-focal or multifocal). In combination with the clinical findings, the three groups were formed.

In the first group, namely, the bone marrow edema (“BME”) class, we placed forty-one (41) subjects (average age 25.5 y) diagnosed with erythropoiesis of bone marrow. These patients were young people and they had more than one focal point of high signal intensity (multifocal) in the presented bones (femur, tibia). They did not have findings of OA or injury of the knee joint.

In the second group, namely, the injured (“INJ”) class, we assigned thirty-seven (37) patients (average age 35.4 y) that had suffered recent injury and bone edema-like lesions, but which did not have findings of OA.

In the third group, namely, the osteoarthritis (“OST”) class, we placed forty-three (43) subjects (average age 61.8 y) with no history of recent injury, but with clinical findings (i.e., chondropathy, mediastinal stenosis, and osteophytes) of OA. In the case of recent injury, MRI examination repetition was conducted to confirm diagnosis.

Differentiation among the three groups is critical to providing accurate diagnosis, appropriate care, optimizing outcomes, preventing complications, and ensuring the efficient use of healthcare resources. Misdiagnosis can lead to inappropriate treatment, delayed management, and potentially worse outcomes. Precise differentiation can prevent excessive medication usage. It allows for tailored, patient-centered care that addresses the specific needs and characteristics of each condition.

### 2.2. MRI Imaging Techniques and ROIs Delineation

All knee examinations were performed using a 1.5 T scanner (SIGNA HDX Twin Speed, GE Healthcare, Chicago, IL, USA) and the General Electric four-channel matrix knee coil. Two sequences were employed: the fast-spin echo (FSE) proton density (PD-FSE) with fat suppression (FS) and the short tau inversion recovery (STIR) sequences in sagittal plane.

PD-FSE is a standard MRI sequence that provides detailed anatomical information by exploiting the differences in the density of protons in various tissues within the body. It is particularly useful for evaluating bone marrow because it highlights the contrast between fat and water. STIR is another MRI sequence that is highly effective in evaluating bone marrow. STIR imaging is a fat-suppressed sequence that is specifically designed to suppress the signal from fat and highlight the signal from water and other soft tissues. The parameters for PD-FSE and STIR sequences are presented in Table 1.

**Figure 1:**
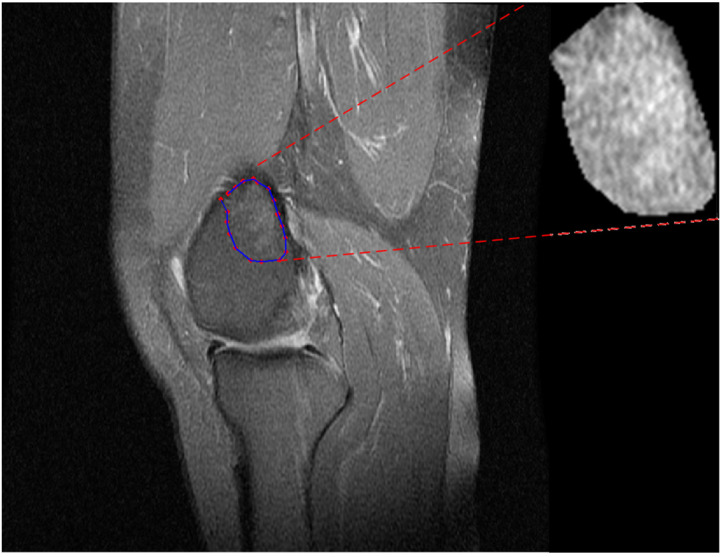
An instance of ROI delineation from bone marrow edema like lesion.

For each case and for each sequence, a region of interest (ROI) was manually delineated by an expert radiologist (A.T.) and an experienced radiographer (E.L.) employing a software program developed for the purposes of the present study. The software provided the radiologist with a series of DICOM MR images and the capability to select appropriate images and to delineate and eventually segment ROIs. Figure 1 shows a snapshot of delineation (bone marrow edema case) in the PD-FSE sequence and the corresponding ROI.

**Figure 2:**
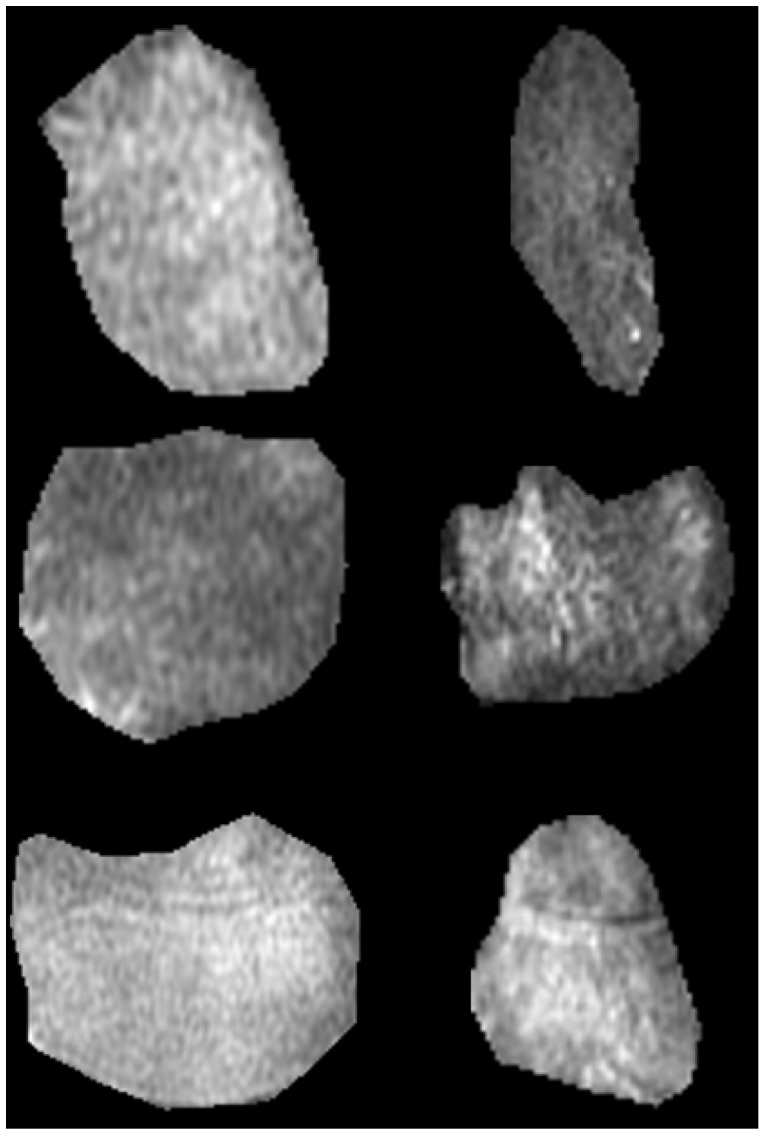
Samples of region of interest for the three categories and two MRI sequences; bone marrow edema (first row), injured (middle row), and osteoarthritis (last row); PD-FSE (left column) and STIR (right column).

Finally, we extracted two ROIs for each case, one corresponding to STIR and one to the PD-FSE sequences accordingly. Figure 2 presents instances from the acquired ROIs according to pathological condition and MRI sequence protocol.

### 2.3. Extraction of Radiomic Descriptors

We computed eighty-one (81) descriptors from a single ROI, encoding texture information and employing a large spectrum of textural features as follows:

This included seventeen (17) descriptors from first-order statistics [22], describing the shape of distribution of pixel intensities in the ROI. These were histogram-based features (such as mean intensity, standard deviation, skewness, kurtosis, median, percentiles, range, interquartile range, and intensity-based entropy).

Fifty-four (54) descriptors were calculated from the second-order statistics, describing the statistical inter-relationships between neighboring pixels.

- Twenty-two (22) of these were calculated based on the gray-level co-occurrence matrix (GLCM) [22,23,24,25,26] (angular second moment, contrast, correlation, sum of squares, inverse difference moment, sum average, sum variance, sum entropy, entropy, difference variance, difference entropy, information measure correlation 1, information measure correlation 2, maximal correlation coefficient, joint maximum, joint average, difference average, inverse difference, normalized inverse difference, autocorrelation, cluster shade, and cluster prominence).
- Sixteen (16) were computed based on the gray-level run-length matrix (GLRLM) [22,27,28,29,30] (short-run emphasis, long-run emphasis, gray-level non-uniformity, run-length non-uniformity, run percentage, low-gray run emphasis, high-gray run emphasis, short-run low-gray emphasis, short-run high-gray emphasis, long-run low-gray emphasis, long-run high-gray emphasis, normalized gray-level non-uniformity, normalized run-length non-uniformity, gray-level run variance, and run-length variance, run entropy). For GLCM and GLRLM descriptors, the average across four orientations (N, S, E, W) was determined (using 1 pixel offset).
- Sixteen (16) were considered based on the gray-level size zone matrix (GLSZM) [22,31] (small-zone emphasis, large-zone emphasis, low-gray-level zone emphasis, high-gray-level zone emphasis, small-zone low-grey emphasis, small-zone high-grey emphasis, large-zone low-grey emphasis, large-zone high-grey emphasis, gray-level non-uniformity, normalized gray-level non-uniformity, zone-size non-uniformity, normalized zone-size non-uniformity, zone percentage, grey-level zone variance, zone-size variance, zone-size entropy)

Six (6) descriptors were calculated based on Tamura descriptors [32] (Tamura coarseness 1, 2, 3, 4, contrast and roughness).

Four (4) descriptors were computed from the local binary pattern (LBP) [33] (mean, median, number of peaks, uniformity)

### 2.4. Statistical and Discriminant Analysis

We studied plausible alterations of textural descriptors amongst pathological conditions based on the statistical comparisons amongst the three groups (as BME vs INJ vs OST).

Initially, we assessed the normality distribution of each one of the features by means of the Jarque–Bera statistical test [34]. The Jarque–Bera test is a statistical test used to assess whether a dataset follows a normal distribution. Next, for those features that followed normal distribution, we employed the ANOVA test to evaluate the existence of statistically significant differences. ANOVA tests the null hypothesis that all group means are equal against the alternative hypothesis that at least one group mean is different. For the features that did not follow normal distribution, we utilized the Kruskal–Wallis test [35] to identify statistically significant differences, and we reported the median (IQR). The Kruskal–Wallis test is a non-parametric statistical test used to determine whether there are statistically significant differences between the medians of three or more independent groups or samples.

Moreover, we performed the multiple comparison test [36] as a post hoc test. Multiple comparison tests, also known as post hoc tests or post hoc analyses, are a group of statistical tests used after ANOVA, Kruskal–Wallis, or other omnibus tests to determine which specific group means are significantly different from each other. These tests are essential in situations where you have conducted ANOVA, Kruskal–Wallis or a similar test and found a significant difference among groups. The goal of multiple comparison tests is to identify which pairs of groups are responsible for this significant difference. To correct the significance level discrepancy that emerged on account of multiple tests, we employed the Benjamini and Hochberg false discovery rate (BH-FDR) [37,38]. Benjamini and Hochberg testing is a statistical method used to control the false discovery rate in multiple hypothesis testing.

Moreover, for the purposes of applying discriminant analysis on the data we employed the algorithms of the python’s Scikit-Learn (sklearn) open-source machine learning library [39]). First, we ordered the calculated radiomics’ descriptors (features) from the PD-FSE and STIR protocols in accordance with their classification importance by employing the sklearn’s feature_importances_ function. Next, we chose the first twenty (20) most highly important features and we formed all the plausible features’ combinations, with up to a maximum of five features per combination. We used each feature combination to design the classification model and we evaluated its classification accuracy. We tested sixteen sklearn classification models [39]. To test the generalization performance of each designed classification model, data were first augmented by applying the sklearn’s resample function and next the three classes were equalized by applying the python’s imbalanced-learn’s SMOTE (Synthetic Minority Over-sampling Technique) function.

For evaluation purposes, data were randomly split into train–validation (70%) datasets and test (30%) datasets using the sklearn’s train_test_split function to have an estimation of error variation. The train–validation (70%) dataset was used to design the classification model, based on the stratified 10-fold cross-validation method. The latter was employed for tuning hyperparameters and assessing the model’s performance during training. The trained model, so far fine-tuned, was used to classify the independent test set (30%). We use the test set to determine how well the model will perform on unseen data. The above process was repeated 10 times; each time, the evaluation metrics of the test set were recorded. Finally, the means of the evaluation metrics were recorded to provide an unbiased estimate of the model’s generalization performance on unseen data. The whole procedure was used to assess the performance for each one of the sixteen classification models.

In this way we found the best classification model providing the highest classification accuracy to the test dataset data. Three basic evaluation metrics were employed on the classified test datasets:

The overall accuracy as $Acc=\frac{TP+TN}{TP+FP+FN+TN}$, the true positive rate as $TPR=\frac{TP}{TP+FN}\;$, and the area under the ROC curve as *AUC*.

## 3. Results and Discussion

In total, two hundred and forty-two (242) ROIs were extracted, eighty-two (82) accounting for the BME group (forty-one for each MRI sequence), seventy-four (74) accounting for the INJ group (thirty-seven for each MRI sequence) and eighty-six (86) accounting for the OST group (forty-three for each MRI sequence).

Concerning the PD-FSE sequence, of the eighty-one (81) descriptors, fifty-one (51) descriptors appeared to show statistically significant differences (*p* < 0.05). We found thirty-four (34) of the 81 descriptors to follow normal distribution according to the Jarque–Bera test, while the forty-seven (47) remaining did not (*p* < 0.05). Six (6) out of the thirty-four descriptors showed statistically significant differences (*p* < 0.001) using the ANOVA test after correcting using the BH-FDR test. Five (5) out of the forty-seven descriptors sustained statistically significant differences (*p* < 0.001) when employing the Kruskal–Wallis nonparametric test after correcting for significance using the BH-FDR test.

Regarding the STIR sequence, of the eighty-one (81) descriptors, thirty-nine (39) descriptors sustained statistically significant differences (*p* < 0.05). We found that thirty (30) of the 81 descriptors followed normal distribution according to the Jarque–Bera test, while the remaining fifty-one (51) did not (*p* < 0.05). Three (3) out of thirty descriptors showed statistically significant differences (*p* < 0.001) when assessed using the ANOVA test after correcting via BH-FDR testing. Eleven (11) out of the fifty-one descriptors showed statistically significant differences (*p* < 0.001) employing the Kruskal–Wallis nonparametric test, after correcting using the BH-FDR test.

Table 2 and Table 3 present the values for the descriptors extracted from PD-FSE and STIR image-ROIs, respectively, that sustained statistically significant differences, along with the post hoc explanation of pairwise comparison. The values reported as the mean ± standard deviation for features were examined using ANOVA testing, and those as the median (Interquartile range—IQR) were examined via the Kruskal–Wallis test.

**Figure 3:**
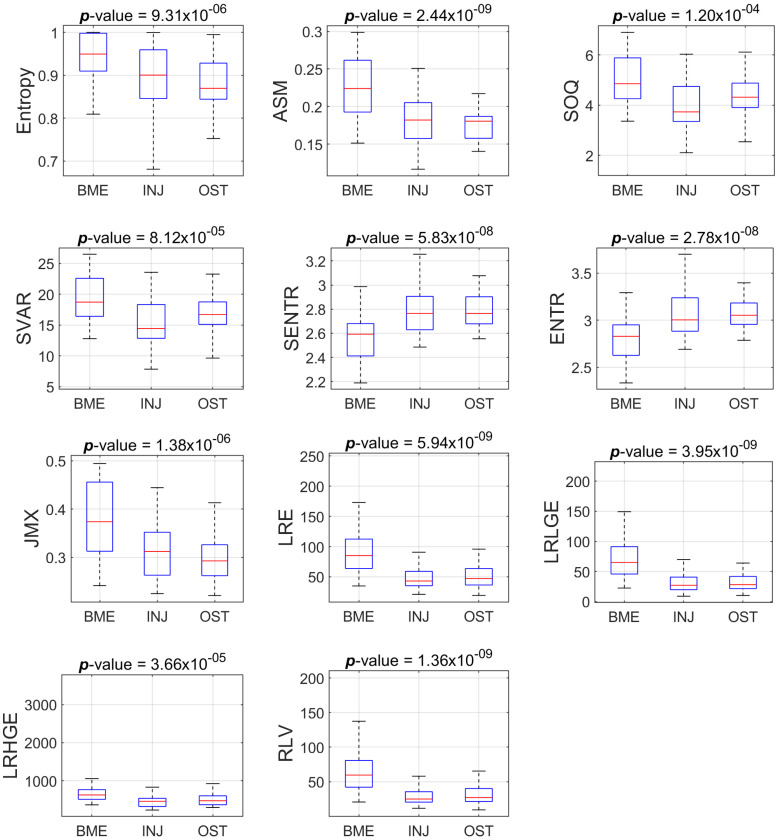
Boxplots of the eleven features that present statistically significant differences amongst the groups (BME vs INJ vs OST) for the ROIs from PD-FSE sequence.

**Figure 4:**
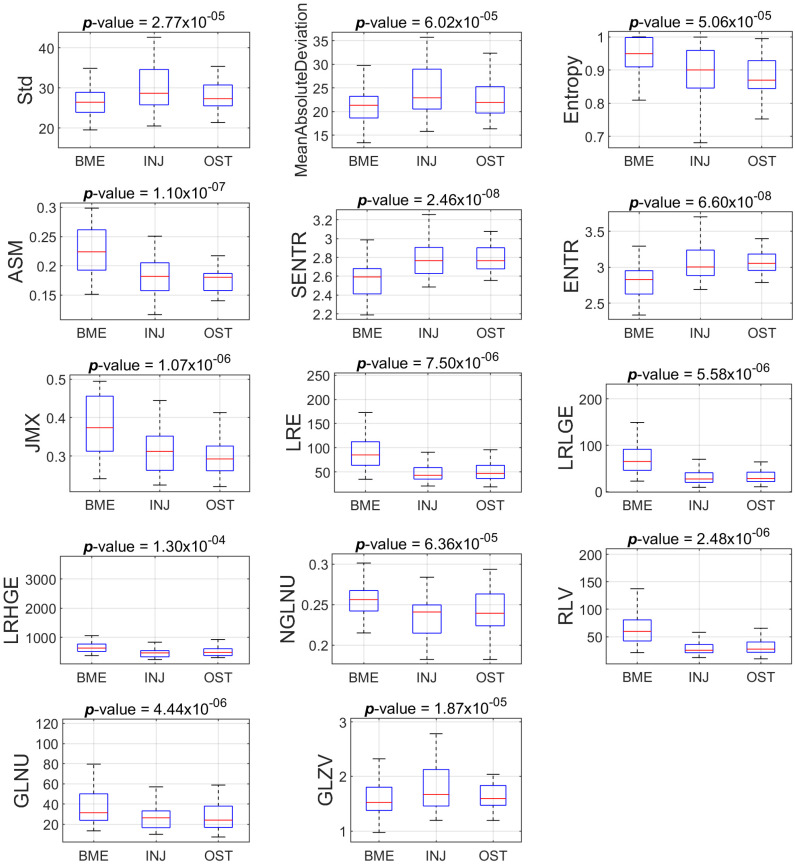
Boxplots of the eleven features that present statistically significant differences amongst the groups (BME vs INJ vs OST) for the ROIs from STIR sequence.

Figure 3 and Figure 4 show the boxplots of the features distribution from BME, INJ and OST groups that present statistically significant differences (*p* < 0.001) concerning the PD-FSE and STIR sequences, respectively.

Table 2 and Table 3 show that there are features with statistically significant differences for one or two pairs of groups, but not for all three pairs of groups. For example, we found statistically significant difference between BME and INJ, as well as between BME and OST with respect to the feature ASM, but we did not find a difference between INJ and OST using the aforementioned feature. In the process of finding some descriptors that, when suitably combined in a regression model as a composite descriptor, may provide a good prediction amongst the group, we tested many different classification models from the Scikit-learn library [39]. The ensemble random forest (ERF) classifier achieved the best performance in terms of Acc, TPR and AUC.

We found five descriptors that appeared in the majority of the descriptors’ combinations for the different evaluation repetitions.

Table 4 presents the metrics values for different validation repetitions. The average metrics scores are Acc: 0.92 ± 0.02, TPR_BME_: 0.94 ± 0.03, TPR_INJ_: 0.91 ± 0.02, TPR_OST_: 0.92 ± 0.03, and AUC: 0.93 ± 0.02.

**Figure 5:**
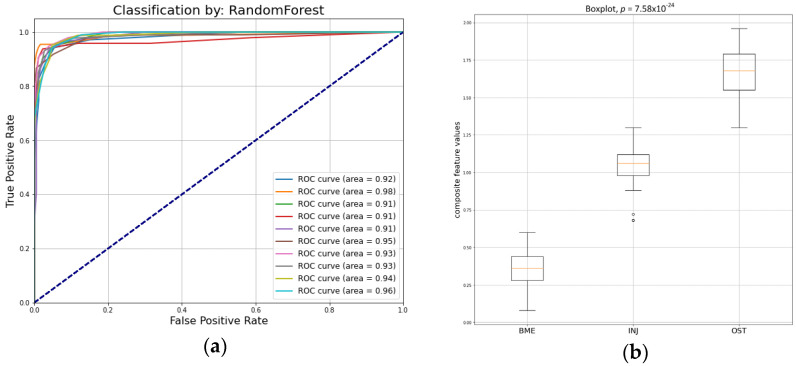
(**a**) ROC curves by the ensemble random forest classifier in test set; (**b**) boxplot for the predicted values of the five combined composite descriptor.

Figure 5a,b present the prediction performance of the five combined features (composite descriptor) in the three categories. These descriptors encode information about first- and second-order statistics, three of them were calculated from the PD-FSE sequence protocol (JMX, LRLGE, RLV), and two were calculated from the STIR sequence protocol (MAD, GLZV).

In particular, the joint maximum (JMX) descriptor is a measure of orderliness in the ROI, considering spatial interrelationships between pixels. High values of the descriptor imply that many identical grey-level neighboring pairs will be located in the ROI. We found higher values in the BME group and smaller values in the OST group when the descriptor was calculated using the PD-FSE protocol ROIs.

The long-run low-gray emphasis (LRLGE) descriptor highlights the existence of large structures along four orientations (N, S, E, W) with low signal intensity, and thus an increased local contrast [29]. We found higher LRLGE values in the BME group and smaller values in the OST group for the ROIs extracted from the PD-FSE protocol images.

Run-length variance (RLV) is a gray-level run-length matrix (GLRLM)-based descriptor that estimates the diversity of structures in the ROI [22]. High values of the descriptor imply a variety in structures in the ROI. We found higher values in the BME group, and smaller values in the OST group when the descriptor was calculated on the basis of PD-FSE protocol ROIs. The importance of structural information extraction has been highlighted in previous work [40].

Mean absolute deviation (MAD) is an intensity-based statistical descriptor and measures the dispersion from the mean [22]. We found smaller deviations from the mean intensity in the BME group, and higher deviations in the INJ group when the descriptor was calculated on the STIR protocol ROIs.

Grey-level zone variance (GLZV) is a grey-level size zone matrix (GLSZM)-based descriptor that quantifies the variance in the number of zones over the grey levels [22]. We found smaller values for the BME group, and higher values for the INJ group when the descriptor is calculated on the STIR protocol ROIs.

Those findings implied deviations in ROI texture as extracted from PD-FSE and STIR sequences. In relation to PD-FSE sequences, we discovered that the BME ROIs exhibit greater local contrast and diversity in structures compared to the OST ROIs, while the INJ ROIs lie in between. Regarding the STIR sequences, the BME ROIs exhibit enhanced homogeneity in signal intensity levels and reduced variation in the local pixel zones compared to the INJ ROIs. On the other hand, the OST ROIs demonstrate intermediate results. Texture variations may originate from the necrosis, fibrosis, and trabeculae differentiations [3] and local subchondral changes in BMLs [8,41].

The division of data into groups was based on radiological findings and this could be considered a limitation of the method. For a more robust approach, arthroscopic findings would be helpful.

Future work may involve identifying and evaluating any textural changes following bone defect fillings. In particular, the integration of biomaterials with the surrounding bone can be analyzed through MRI texture analysis [42]. A successful filling should exhibit a texture that closely resembles the adjacent healthy bone tissue. Furthermore, MRI texture analysis may facilitate the monitoring of integration progress. This might be valuable in order to comprehend how well the filling material acclimates to the mechanical stresses and biological environment of the bone [43]. Changes in texture over time may suggest whether the integration is getting better or whether there are complications such as fibrosis or inflammation. Lastly, MRI texture analysis could be used to compare the integration of diverse filling materials. This might help the selection of appropriate materials for an individual patient and the optimization of treatment protocols.

## 4. Conclusions

In conclusion, under the concept that changes in the subchondral bone microenvironment may be captured by studying textural image properties, we found that the study of bone marrow edema in MRI knee images through texture analysis and machine learning methods holds significant promise in the field of musculoskeletal medicine. Radiomics analysis may provide a non-invasive and quantitative means of assessing the spatial distribution and heterogeneity of bone marrow edema, aiding in its early detection and characterization. As technology and methodologies continue to advance, the integration of radiomic analysis and machine learning methods into clinical practice is likely to play an increasingly vital role in the management of knee joint conditions.

## Figures and Tables

**Table 1:** Sequences parameters for proton density (PD-FSE) fast-spin echo (FSE) with fat suppression and short tau inversion recovery (STIR).

Sequences	PD-FSE	STIR
Parameters		
ΤR (ms)	2200	5675
TE (ms)	30	50
ETL	8	16
Band Width (kHz)	31.25	20.83
Freq	384 × 224	256 × 192
Freq DIR	A/P	A/P
Slice Thickness (mm)	4.0	4.0
Spacing (mm)	0.8	0.8
FOV (mm)	18	18
Slices	19	18
Acquisition Time (min)	2′.17″	2′.22″

**Table 2:** Features sustained statistically significant differences amongst the categories concerning the PD-FSE sequence, after BH-FDR correction.

Feature	BME Mean ± Std or Median (IQR)	INJ Mean ± Std or Median (IQR)	OST Mean ± Std or Median (IQR)	Description of Post Hoc Pairwise Comparison (*p* < 0.001)
Entropy	0.95 (0.09)	0.90 (0.11)	0.87 (0.08)	The mean ranks of groups BME and OST are significantly different
ASM	0.22 (0.07)	0.18 (0.05)	0.18 (0.03)	Two groups have mean ranks significantly different from BME
SOQ	4.86 (1.63)	3.74 (1.39)	4.32 (0.97)	The mean ranks of groups BME and INJ are significantly different
SVAR	18.73 (6.17)	14.45 (5.48)	16.71 (3.65)	The mean ranks of groups BME and INJ are significantly different
SENTR	2.59 (0.27)	2.77 (0.28)	2.77 (0.22)	Two groups have mean ranks significantly different from BME
ENTR	2.83( 0.32)	3.00 (0.35)	3.05 (0.23)	Two groups have mean ranks significantly different from BME
JMX	0.38± 0.08	0.32± 0.06	0.30± 0.05	The mean ranks of groups BME and OST are significantly different
LRE	97.74 ± 49.12	53.51 ± 29.61	51.62 ± 19.99	Two groups have mean ranks significantly different from BME
LRLGE	77.56 ± 47.40	36.40 ± 25.90	33.93 ± 17.51	Two groups have mean ranks significantly different from BME
LRHGE	727.09 ± 497.94	478.65 ± 179.35	553.00 ± 229.49	The mean ranks of groups BME and INJ are significantly different
RLV	70.55 ± 41.60	33.38 ± 21.96	32.01 ± 14.96	Two groups have mean ranks significantly different from BME

**Table 3:** Features sustained statistically significant differences amongst the categories concerning the STIR sequence, after BH-FDR correction.

Feature	BME Mean ± Std or Median (IQR)	INJ Mean ± Std or Median (IQR)	OST Mean ± Std or Median (IQR)	Description of Post Hoc Pairwise Comparison (*p* < 0.001)
Std	29.10 (7.87)	36.58 (8.57)	33.29 (7.71)	Two groups have means significantly different from BME
MAD	23.64 (6.66)	29.40 (8.18)	27.09 (6.30)	Two groups have means significantly different from BME
Entropy	0.96± 0.05	0.90± 0.07	0.92± 0.06	The mean ranks of groups BME and INJ are significantly different
ASM	0.20± 0.05	0.15± 0.03	0.15 ± 0.04	Two groups have mean ranks significantly different from BME
SENTR	2.72 ± 0.23	3.02 ± 0.18	3.00 ± 0.23	Two groups have mean ranks significantly different from BME
ENTR	3.12 ± 0.30	3.50 ± 0.25	3.47 ± 0.32	Two groups have mean ranks significantly different from BME
JMX	0.37 ± 0.08	0.28 ± 0.06	0.30 ± 0.06	Two groups have mean ranks significantly different from BME
LRE	43.60 ± 26.98	22.57 ± 12.06	25.47 ± 16.69	Two groups have mean ranks significantly different from BME
LRLGE	33.96 ± 24.88	14.74 ± 10.28	17.26 ± 12.92	Two groups have mean ranks significantly different from BME
LRHGE	277.32 ± 122.49	195.16 ± 80.30	217.82 ± 132.22	The mean ranks of groups BME and INJ are significantly different
NGLNU	0.23 ± 0.02	0.21 ± 0.02	0.22 ± 0.02	Two groups have mean ranks significantly different from BME
RLV	29.22 ± 20.74	12.80 ± 8.49	14.52 ± 10.82	Two groups have mean ranks significantly different from BME
GLNU^sz^	21.48 ± 10.27	14.00 ± 8.35	12.23 ± 5.76	Two groups have mean ranks significantly different from BME
GLZV	1.7 4 0.50)	2.27 (0.54)	2.08 (0.51)	The means of groups BME and INJ are significantly different

**Table 4:** Classification metrics for different evaluation repetitions in the Test sets.

k-fold	Acc	TPR			AUC
		BME	INJ	OST	
1	0.89	0.89	0.89	0.89	0.92
2	0.96	0.99	0.94	0.94	0.98
3	0.91	0.93	0.90	0.91	0.91
4	0.92	0.90	0.90	0.96	0.91
5	0.91	0.91	0.93	0.89	0.91
6	0.92	0.94	0.87	0.95	0.95
7	0.93	0.94	0.92	0.93	0.93
8	0.93	0.93	0.91	0.94	0.93
9	0.91	0.98	0.90	0.86	0.94
10	0.93	0.96	0.92	0.91	0.96
Average (std)	0.92 (0.02)	0.94 (0.03)	0.91 (0.02)	0.92 (0.03)	0.93 (0.02)

## Data Availability

The data presented in this study are available on request from the corresponding author.
